# Metformin Uniquely Prevents Thrombosis by Inhibiting Platelet Activation and mtDNA Release

**DOI:** 10.1038/srep36222

**Published:** 2016-11-02

**Authors:** Guang Xin, Zeliang Wei, Chengjie Ji, Huajie Zheng, Jun Gu, Limei Ma, Wenfang Huang, Susan L. Morris-Natschke, Jwu-Lai Yeh, Rui Zhang, Chaoyi Qin, Li Wen, Zhihua Xing, Yu Cao, Qing Xia, Yanrong Lu, Ke Li, Hai Niu, Kuo-Hsiung Lee, Wen Huang

**Affiliations:** 1 Laboratory of Ethnopharmacology, Institute for Nanobiomedical Technology and Membrane Biology, Regenerative Medicine Research Center, the State Key Laboratory of Biotherapy and Cancer Center, West China Hospital, Sichuan University, Chengdu, Sichuan, China; 2 Natural Products Research Laboratories, UNC Eshelman School of Pharmacy, University of North Carolina at Chapel Hill, Chapel Hill, North Carolina, United States; 3 Clinical Laboratory, Hospital of University of Electronic Science and Technology of China and Sichuan Provincial People’s Hospital, Chengdu, Sichuan, China; 4 Department of Integrated Traditional Chinese and Western Medicine, West China Hospital, West China Medical School, Sichuan University, Chengdu, Sichuan, China; 5 Department of Cardiovascular Surgery, West China Hospital, Sichuan University, Chengdu, Sichuan, China; 6 Department of Pharmacology, College of Medicine, Kaohsiung Medical University, Kaohsiung, Taiwan; 7 Department of Emergency Medicine, West China Hospital, Sichuan University, Chengdu, Sichuan, China; 8 Key Laboratory of Transplant Engineering and Immunology, Ministry of Health, West China Hospital, Sichuan University, Sichuan University, Chengdu, Sichuan, China; 9 College of Mathematics, Sichuan University, Chengdu, Sichuan, China; 10 Chinese Medicine Research and Development Center, China Medical University and Hospital, Taichung, Taiwan

## Abstract

Thrombosis and its complications are the leading cause of death in patients with diabetes. Metformin, a first-line therapy for type 2 diabetes, is the only drug demonstrated to reduce cardiovascular complications in diabetic patients. However, whether metformin can effectively prevent thrombosis and its potential mechanism of action is unknown. Here we show, metformin prevents both venous and arterial thrombosis with no significant prolonged bleeding time by inhibiting platelet activation and extracellular mitochondrial DNA (mtDNA) release. Specifically, metformin inhibits mitochondrial complex I and thereby protects mitochondrial function, reduces activated platelet-induced mitochondrial hyperpolarization, reactive oxygen species overload and associated membrane damage. In mitochondrial function assays designed to detect amounts of extracellular mtDNA, we found that metformin prevents mtDNA release. This study also demonstrated that mtDNA induces platelet activation through a DC-SIGN dependent pathway. Metformin exemplifies a promising new class of antiplatelet agents that are highly effective at inhibiting platelet activation by decreasing the release of free mtDNA, which induces platelet activation in a DC-SIGN-dependent manner. This study has established a novel therapeutic strategy and molecular target for thrombotic diseases, especially for thrombotic complications of diabetes mellitus.

Worldwide, 415 million people, or 1 in 11 adults, are estimated to have diabetes mellitus (DM). Approximately 5.0 million people died from DM in 2015, which is equivalent to one death every six seconds^1^,^2^,^3^. Thrombosis is the leading cause of morbidity and mortality in patients with DM, with a reported 65% of diabetic patients eventually dying from thrombotic diseases^4^,^5^,^6^. However, an increased understanding of the mechanisms of thrombogenesis has led to a new era in the discovery of antithrombotic agents that target many of the key steps in blood coagulation and platelet activation^7^. Metformin is widely used as the first-line therapy for type-2 diabetic patients. It has been reported that metformin reduced mortality and diabetes-associated thrombotic complications^8^,^9^,^10^,^11^,^12^. However, its mechanism of action is largely unknown with respect to thrombosis prevention, and further investigation is merited.

In 2000, two independent publications showed that metformin protects mitochondrial function by inhibiting complex I in the mitochondrial respiratory chain^13^,^14^. In 2010, it was reported that metformin use may decrease mortality among patients with diabetes and atherothrombosis^9^. Numerous studies have reported that mitochondrial function is associated with platelet activation and thrombosis^15^,^16^,^17^,^18^. For example, Brownlee and colleagues demonstrated that hyperglycaemia induces mitochondrial hyperpolarization in normal platelets, resulting in the augmentation of reactive oxygen species (ROS) generation and subsequent activation^16^. Cardenes *et al*. found that mitochondrial alterations cause platelet activation *in vitro* and *in vivo*^17^, while Boudreau *et al*. reported that activated platelets can release mitochondria^18^. However, it is not known whether metformin is associated with platelet activation. We hypothesized that metformin can inhibit platelet activation and platelet-activated blood coagulation by preventing platelet mitochondrial dysfunction and release.

## Results

### Metformin suppresses mtDNA release and mitochondrial dysfunction of activated platelets

**Figure 1:**
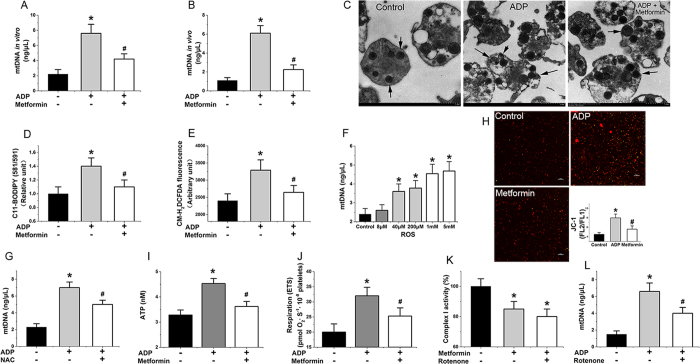
Metformin suppresses the release of platelet mtDNA via inhibition of complex I. (**A**,**B**) Metformin influences mtDNA escape from activated platelets *in vitro* (metformin: 1 mM, 6 h) and *in vivo* (metformin: 400 mg/kg/d, 7 d). (**C**) Transmission electronic microscopy imaging of platelets and mitochondria (black arrows) treated with metformin or without. (**D**) C11-BODIPY 581/591 (lipid peroxidation sensor), (**E**) ROS(CM-H_2_DCFDA fluorescence), (**I**) ATP, (**H**) hyperpolarization (JC-1 fluorescence ratio, FL2/FL1) ΔΨ, (**J**) mitochondrial routine respiration, (**K**) complex I values in activated platelets treated with metformin (metformin: 1 mM, 6 h). (**F**) ROS (hydrogen peroxide, 6 h) induces mtDNA escape from platelets. (**G**) NAC reduce mtDNA release from platelets (NAC: 10 mM, 6 h). (**L**) Low concentrations of rotenone reduce mtDNA release from platelets (rotenone: 12.5 nM, 6 h). Data are expressed as mean ± SD. *n* = 8. *P < 0.05 vs control, ^*#*^*P* < 0.05 vs ADP.

We first determined the amounts of extracellular mtDNA detected from ADP-activated platelets *in vitro* (Fig. 1A, metformin: 1 mM, 6 h, 37 °C) and *in vivo* (Fig. 1B, metformin: 400 mg/kg/d, 7 d) in the absence and presence of metformin with qPCR assay. As expected, metformin inhibited mtDNA release from activated platelets (Fig. 1A,B). Furthermore metformin also inhibited mtDNA release from arachidonic acid (AA)- and thrombin-activated platelets (Supplementary Fig. 1A). However, the mechanism behind these inhibitory effects of metformin is poorly recognized. As shown in Supplementary Fig. 1D, metformin has no effects on apoptosis in platelets. This study eliminated the possibility that the changes of extracellular mtDNA level are because of the changes of apoptotic bodies. Our results suggest that metformin prevented platelet membrane damage in activated platelets (Fig. 1C), Activated platelets treated with metformin also showed lowered lipid peroxidation levels as measured by Lipid Peroxidation Sensor, BODIPY^®^ 581/591 C11 (Fig. 1D). It has been reported that cell membranes are highly susceptible to injury by an over-load of ROS, which then cause enhanced lipid peroxidation leading to damage of the bio-membrane system^19^,^20^. In our study, we observed an elevated level of ROS in ADP-activated platelets as detected by CM-H_2_DCFDA fluorescence (Fig. 1E), but a lower level of ROS production when metformin was added (Fig. 1E). We found that metformin also reduced level of platelet mitochondrial ROS production (Supplementary Fig. 1F). Furthermore, we identified a hydrogen peroxide-induced increase in free mtDNA from activated platelets (Fig. 1F), while N-acetylcysteine (NAC), an ROS inhibitor, also inhibited mtDNA release (Fig. 1G). We further determined the hyperpolarization level of the mitochondrial membrane potential (Fig. 1H), intracellular ATP level (Fig. 1I) and mitochondrial routine respiration (Fig. 1J), and found that these three key indexes for evaluating mitochondrial function were improved from mitochondrial dysfunction with metformin (1 mM, 6 h) treatment in activated platelets. The mitochondrial electron transport chain (ETC) is an important target that regulates mitochondrial function, including mitochondrial membrane potential, ROS, and ATP levels^19^. Our study found that metformin inhibited complex I activity of the platelet mitochondrial respiratory chain (Fig. 1K). In addition, other complexes of the mitochondrial respiratory chain were also evaluated. However, we did not observe any statistical differences in complex activity between metformin treatment and non-treatment. The low concentrations of rotenone, a known mitochondrial complex I inhibitor, had similar effects in this assay (Fig. 1K,L) and, as expected, also inhibited mitochondrial release (Fig. 1L). The effect of rotenone on platelet mtDNA release should be looking at the impact of rotenone dose response, high concentrations of rotenone cause platelet mitochondrial dysfunction, apoptosis and mtDNA release. As shown in Supplementary Fig. 1B, high concentrations of metformin also increase platelet mtDNA release. Thus, our results indicated that metformin lowered mtDNA release from activated platelets and suppressed mitochondrial dysfunction via inhibition of complex I in the ETC.

### mtDNA-induced platelet activation may through DC-SIGN-dependent pathway

**Figure 2:**
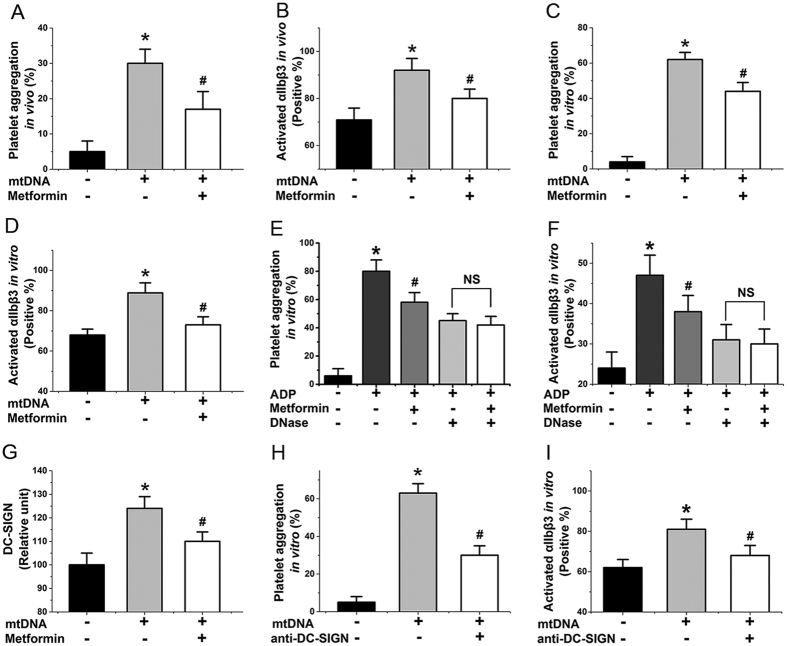
mtDNA induced platelet activation may through DC–SIGN dependent pathway. (**A**,**C**) mtDNA affects aggregation of platelets with or without metformin treatment *in vivo* (mtDNA: 50 μg/kg/d, 7 d) and *in vitro* (mtDNA: 40 ng/μL, 6 h). (**B**,**D**) mtDNA influences αIIbβ3 level with or without metformin treatment *in vivo* and *in vitro*. (**E**,**F**) DNase decreases inhibition of metformin on platelet aggregation and αIIbβ3 level induced by ADP *in vitro* (Pre-incubated for 0.5 h at 20 μg/mL DNase I). (**G**) mtDNA increases DC-SIGN expression *in vitro*. (**H**,**I**) Anti-DC-SIGN (Pre-incubated for 0.5 h at 25 μg/mL) agent decreases platelet aggregation and αIIbβ3 level induced by mtDNA *in vitro*. Data are expressed as mean ± SD. *n* = 8. *P < 0.05 vs control, ^*#*^*P* < 0.05 vs mtDNA.

Overall, our above studies showed that metformin protected against mitochondrial dysfunction by inhibiting complex I, hyperpolarization, ROS production, and ROS-induced membrane damage, and subsequently prevented mtDNA release from activated platelets. However, because it is unclear whether mtDNA induces platelet activation, we also performed additional related experiments. We found that mtDNA caused increased levels of platelet aggregation (Fig. 2A,C) and αIIbβ3 expression (Fig. 2B,D) *in vivo* (mtDNA: 50 μg/kg/d, 7 d) and *in vitro* (mtDNA: 40 ng/μL, 6 h, 37 °C), while the addition of metformin reduced these levels (Fig. 2A–D). To further confirm the importance of mtDNA in metformin inhibited platelet activation it would been useful to demonstrate that elimination of DNA using a DNase eliminated the signal. As expected, DNase prevents inhibition of metformin on platelet activation (Fig. 2E,F). Furthermore, we found that the level of a certain protein, with a comparable weight to that of DC-SIGN, increased when we incubated platelets with mtDNA (Fig. 2G). By using a co-immunoprecipitation assay with anti-DC-SIGN antibodies, we confirmed that this protein was DC-SIGN^21^. Platelets express this surface molecule, which acts as a critical receptor in further platelet activation^21^,^22^,^23^, but had not been linked previously to mtDNA-induced platelet activation. Less DC-SIGN was expressed in the presence of metformin (Fig. 2G). Anti-DC-SIGN antibody (neutralizing antibodies against DC-SIGN, 25 μg/mL, 0.5 h) decreased mtDNA-induced platelet aggregation and αIIbβ3 expression (Fig. 2H,I). Taken together, our study has been the first to reveal that mtDNA released from activated platelets can act as a cellular damage factor and subsequently induce platelet activation may in a DC-SIGN-dependent manner. Next, we considered to confirm that mtDNA induces platelet activation through DC-SIGN with knockout approaches.

### Metformin inhibits platelet activation *in vivo* and *in vitro*

**Figure 3:**
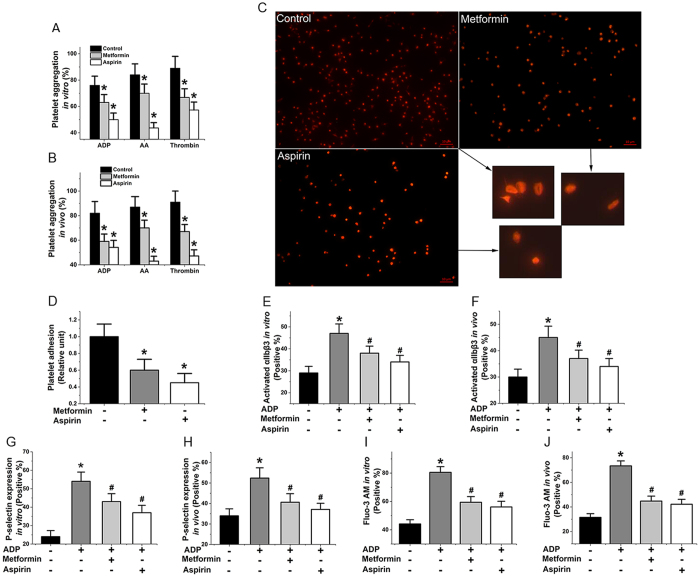
Metformin inhibits platelet activation *in vivo* and *in vitro*. (**A**,**B**) Influence of metformin on aggregation of platelets induced by ADP, AA, and thrombin *in vitro* (metformin: 1 mM, 6 h) and *in vivo* (metformin: 400 mg/kg/d, 7 d). (**C**,**D**) Effect of metformin on platelet adhesion to collagen-coated surfaces (phalloidin-labelled platelets). (**E**,**F**) αIIbβ3 expression in ADP-activated platelets with metformin *in vitro* and *in vivo*. (**G**,**H**) P-selectin expression in ADP-activated platelets with treatment *in vitro* and *in vivo*. (**I**,**J**) Influence of metformin on the elevation of cytosolic calcium levels in ADP-stimulated platelets *in vitro* and *in vivo*. Data are expressed as mean ± SD. *n* = 9–10. *P < 0.05 vs control, ^#^*P* < 0.05 vs ADP.

To further assess the effect of metformin on platelet activation, we assessed the drug’s effect on aggregation of platelets induced by ADP, AA, and thrombin *in vitro* (Fig. 3A, metformin: 1 mM, 6 h, 37 °C) and *in vivo* (Fig. 3B, metformin: 400 mg/kg/d, 7 d). The results showed that metformin significantly suppressed platelet aggregation in all assays (Fig. 3A,B). We also found that three key indicators of platelet activation, αIIbβ3 expression (Fig. 3E,F), P-selectin (Fig. 3G,H), and cytosolic calcium (Fig. 3I,J), were decreased *in vitro* and *in vivo* in ADP-activated platelets in the presence of metformin. The effects were comparable with those of aspirin used as a positive control (Fig. 3D–J). The αIIbβ3 expression that key indicator of platelet activation also were decreased in AA, and thrombin-activated platelets in the presence of metformin (Supplementary Fig. 2A). Furthermore, because thrombosis is known to be mediated by adhesion, activation, and aggregation of platelets^24^, we evaluated the effects of metformin on platelet adhesion and activation on collagen-coated surfaces. The data showed that metformin effectively inhibited these processes (Fig. 3C,D).

### Metformin inhibits the formation of arterial and venous thrombosis in animals

**Figure 4:**
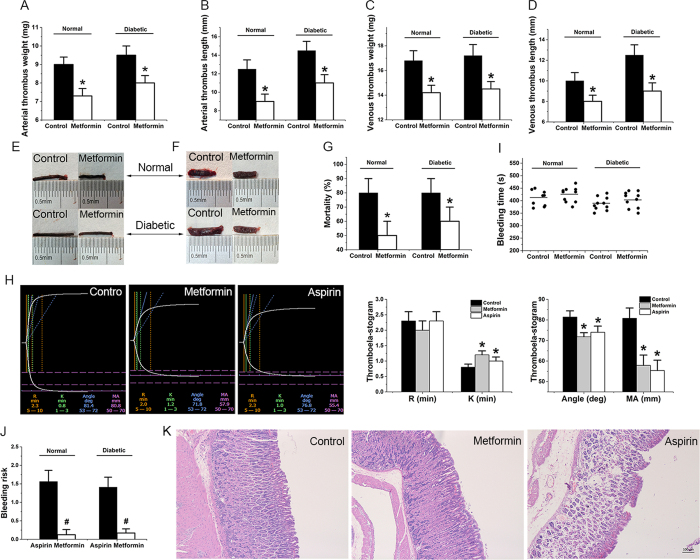
Metformin inhibits formation of FeCl_3_-induced carotid arterial thrombosis and partial inferior vena cava ligation induced venous thrombosis in animals. Decreases weight (**A,E**) and length (**B,E**) of arterial thrombus in diabetic and normal rats (pre-treated with 400 mg/kg/d metformin for 7 d). Decreases weight (**C,F**) and length (**D,F**) of venous thrombus in normal and diabetic rats. (**G**) Reduces mortality from pulmonary thromboembolism in diabetic and normal mice (metformin: 400 mg/kg/d, 7 d). (**H**) Thromboelastogram of whole blood from rats treated with or without metformin. (**I**) Does not significantly prolong bleeding times in normal and diabetic C57/BL6 mice (metformin: 400 mg/kg/d, 7 d). (**J**) Reduces bleeding risk (increased bleeding time %/inhibited thrombosis %) in diabetic and normal rats compared with the use of aspirin. (**K**) Metformin decreases the incidence of gastric ulcer in normal rats compared with the use of aspirin (metformin: 400 mg/kg/d, 60 d. Aspirin: 30 mg/kg/d, 60 d). Data are expressed as mean ± SD. *n* = 9–10. *P < 0.05 vs control, ^#^*P* < 0.05 vs aspirin.

In addition, we evaluated the effect of metformin on thrombus (metformin: 200 mg/kg twice a day for 7 days)^25^,^26^, particularly on diabetic rat thrombus, since metformin is an antidiabetic drug^6^. Metformin showed similar effects on preventing arterial (Fig. 4A,B,E) and venous (Fig. 4C,D,F, Supplementary Fig. 3A,B) thrombosis in normal and diabetic rat models. The metformin treatment significantly decreased the incidence of pulmonary embolism (Fig. 4G), reduced the weight and length of arterial and venous thrombus (Fig. 4A–F), prolonged the average time to occlusive thrombosis in FeCl_3_-induced inferior vena cava and carotid arteries injury (Supplementary Fig. 3C,D), and improved blood viscosity (Supplementary Table 1). These results suggest that metformin may have great potential as an anti-thrombotic agent and are consistent with published reports stating that metformin decreases cardiovascular events and mortality in diabetic patients^27^,^28^,^29^. Our studies also demonstrate that metformin reduces the amount of plasma mtDNA and platelet DC-SIGN expression in FeCl_3_-induced carotid arterial thrombosis and partial inferior vena cava ligation induced venous thrombosis model (Supplementary Fig. 3E–H). From analysis of a thromboelastogram (TEG)^30^, we found that metformin treatment significantly improved indexes for viscoelastic properties of the developing clot, thrombogenesis, and platelet activation (Fig. 4H). However, metformin treatment appeared not to have a significant influence on prothrombin time (PT), activated partial thrombin time (APTT), fibrinogen (FIB), thrombin time (TT), coagulation factors II, V, VII–XII, clot weight, and platelet counts (Supplementary Fig. 2B–D, Supplementary Table 2). Furthermore, the metformin treatment did not significantly prolong bleeding time in either normal or diabetic rats (Fig. 4I). These findings are notable, because a known major limitation of current antithrombotic therapies is their inability to separate thrombotic events from bleeding occurrences^31^. And the mice treated with metformin did not show any spontaneous bleeding and had far less bleeding risk than those treated with aspirin (Fig. 4J), the most commonly used oral antithrombotic drug. Our results in mice are consistent with the fact that bleeding side effects do not generally occur with clinical metformin treatment^12^. Our results also showed that, compared with aspirin^32^, metformin treatment (metformin: 200 mg/kg twice a day for 60 days) led to decreased incidence of gastric ulcer (Fig. 4K). In our studies, gastric ulcers developed in two mice (2/10) in the aspirin-treated group (aspirin: 15 mg/kg twice a day for 60 days), but none appeared in mice (0/10) in the metformin-treated group. These data suggest that metformin can prevent both venous and arterial thrombosis by inhibiting platelet activation, notably without a significant risk of bleeding.

## Discussion

Metformin, an insulin-sensitizer, may improve vascular function and several physiologic abnormalities related to insulin resistance with fewer reported side-effects in patients with type 2 DM^10^. Literature reports have connected metformin with reduced mortality and diabetes-associated cardiovascular and cerebrovascular events in diabetic patients^8^,^9^,^10^,^11^. However, it is not known whether metformin is effective against thrombosis in both diabetic and normal individuals, and further investigation is merited to determine its precise mechanism of action with respect to thrombosis prevention. One advantage of normal animal thrombotic models, as opposed to diabetes-associated studies, is that the contribution of metformin can be assessed independently of other cardiovascular risk factors, such as hypercholesterolemia, hyperglycemia, obesity, and hypertension^33^. By using models of carotid artery and inferior vena cava thrombosis, we found that thrombus formation was strongly impaired in metformin-treated diabetic and normal rats. We also demonstrated that metformin-treated mice were more resistant to pulmonary thromboembolism than control mice. Normal activity of blood coagulation requires platelets as a critical factor. Studies have shown that platelets are involved in many biological clotting processes, including detection of vascular lesions, coagulation cascade startup, and activation of other platelets^15^. Additional studies presented here provided evidence that, in the presence of metformin, platelet prothrombinase activity was markedly impaired. This finding is consistent with the observed decrease in αIIbβ3 expression, P-selectin, and cytosolic calcium of platelets preincubated with metformin. Our results also indicated that the platelet adhesive function was reduced after administration of metformin. However, metformin treatment appeared to have no significant influence on coagulation factors and clot weight.

Antiplatelet therapy has been effective in reducing the mortality and morbidity of cardiovascular disease, the most common cause of death in developed countries. However, current antiplatelet agents approved by the US Food and Drug Administration have serious side effects, including bleeding episodes, gastrointestinal toxicity, neutropenia and thrombocytopenia. As stated previously, a recognized limitation of current antiplatelet therapies is their inability to separate thrombotic events from bleeding occurrences^31^,^33^,^34^. The impact of metformin on hemostatic function is best exemplified by our tail bleeding time studies and chronic-type gastric ulcer evaluation. Intriguingly, metformin-treated mice did not show any spontaneous bleeding, abnormal tail bleeding times, or gastrointestinal toxicity (without gastric ulcer occurrences). Our mouse studies also demonstrated that metformin does not have deleterious effects on platelet properties and, therefore, suggested that long-term medication would not lead to the negative effects of induced platelet death or altered platelet counts. In this study, we have demonstrated that metformin uniquely prevents both venous and arterial thrombosis with no significant prolonged bleeding time by inhibiting platelet activation. Our work presented herein suggests that further investigation on metformin could lead to a novel antiplatelet therapy with fewer side-effects. Increased understanding of metformin’s antithrombotic activity should lead to the development of new antiplatelet drugs that are potentially more effective with minimal side effects. Such new therapies targeting the underlying mechanism for platelet dysfunction are urgently warranted.

Some indications as to the role mitochondria play in platelet function have been elucidated^15^,^16^,^17^. Early reports primarily examined the role that mitochondria play in meeting the energy demands required for platelet aggregation and granule release^16^. For example, Barile *et al*. pointed out that inhibition of mitochondrial respiration in platelets could suppress platelet-stimulated blood coagulation^15^. Interestingly, previous work by Boudreau and colleagues demonstrated that activated platelets can release mitochondria^18^. Previously, various studies indicated that metformin has variable effects on hepatic mitochondrial function by inhibiting complex I in the mitochondrial respiratory chain^13^,^14^. While it was not determined whether metformin can protect against activated platelet-induced mitochondrial dysfunction or prevent mitochondria release from activated platelets, we hypothesized that metformin can inhibit platelet activation and platelet-activated blood coagulation in this manner. Consistent with our hypothesis, decreased extracellular mtDNA was detected with quantitative real-time PCR in metformin-treated platelets. As shown in Supplementary Fig. 1D, metformin had no significant effect on apoptosis of platelets. Therefore it was conclude that the lowered level of extracellular mtDNA may not be caused by the variation of platelets apoptotic bodies. Our observations also indicated that metformin inhibited mtDNA release by preventing membrane damage, and mitochondrial dysfunction in activated platelets. However, the mechanism behind these inhibitory effects of metformin is poorly recognized and deserves further investigation. Membranes are redox-sensitive, and an increased generation of ROS is one mechanism for membrane damage^19^. Our results showed that lower levels of ROS were produced when platelets were preincubated with metformin. We subsequently explored several possible mechanisms for metformin-induced decreased accumulation of ROS in activated platelets. We first considered decreased NADPH oxidase in platelets as a potential mechanism, because decreased NADPH oxidase has been associated with platelet activation^33^. However, we did not observe any differences in platelet NADPH oxidase levels between metformin treatment and untreated control (Supplementary Fig. 1G). Glutathione peroxidase-1 (Gpx1) was also evaluated, as decreased erythrocyte Gpx1 activity is associated with increased cardiovascular risk in patients with coronary artery disease; however, no change was seen (Supplementary Fig. 1H). We next considered the potential role of superoxide dismutase as an enzymatic source of elevated ROS in platelets. Because ROS can be generated from superoxide by superoxide dismutase^33^, metformin-associated decreased generation of ROS in platelets could result from decreased conversion of superoxide to H_2_O_2_ by superoxide dismutase. However, we consider this possibility to be unlikely, because we also did not detect any significant change of superoxide dismutase (*Sod1*) in platelets from metformin treatment (Supplementary Fig. 1I).

ETC is an important target that regulates ROS levels and mitochondrial function, including mitochondrial membrane potential, respiration and ATP levels^19^. As expected, our findings that complex I in the ETC was inhibited in metformin-treated platelet were consistent with the observations of Piel *et al*. in human platelets^35^. In this study, we also found that intracellular ATP level, mitochondrial routine respiration, and hyperpolarization level of the mitochondrial membrane potential, three key indexes for evaluating mitochondrial function, were improved with metformin treatment in ADP-stimulated platelets. These findings suggest that the decrease in platelet ROS is caused by decreased activity of mitochondrial complex I. In agreement with this hypothesis, we found that preincubation of platelets with low concentrations of rotenone, a mitochondrial complex I inhibitor, resulted in a significant decrease in ADP-induced activation of αIIbβ3 (data not shown). We also observed that low concentrations of rotenone decreased mtDNA release from ADP-induced activated platelets. Meanwhile, high concentrations of rotenone caused platelet mitochondrial dysfunction, apoptosis, and mtDNA release. Similarly to rotenone, the effect of metformin on platelet mtDNA release should be looking at the impact of metformin dose response, high concentrations of metformin increased mtDNA release by increasing platelet apoptosis, low concentrations of metformin inhibited mtDNA release by protecting mitochondrial function. Taken together, our results indicated that metformin lowers mtDNA release from activated platelets and suppresses mitochondrial dysfunction via inhibition of complex I in the ETC.

Increasing evidence demonstrates that mtDNA plays key roles in immune and inflammatory responses and their related diseases^27^,^36^,^37^,^38^,^39^, and our results indicated that metformin inhibits mtDNA release as well as platelet activation. However, because it is unclear whether mtDNA induces platelet activation, additional related experiments were performed. We examined the effect of mtDNA on platelet pro-coagulant activity, and found that mtDNA-treated platelets can support efficient platelet activation *in vitro* and *in vivo*. However, the nature of the downstream pathways for mtDNA-mediated platelet formation remains largely unknown. DC-SIGN, a type II membrane protein and C-type lectin receptor that binds high-mannose-containing glycoproteins, was originally cloned from a placental library via its ability to bind the glycan-rich HIV-1 envelope without the assistance of the classic CD4 virus receptor^22^. Although previous studies have shown that DC-SIGN interacts with platelet activation^21^, they were not then linked to mtDNA-induced platelet activation. When we incubated platelets with mtDNA, the level of a certain protein was increased. Next, we confirmed that this protein was DC-SIGN by using a co-immunoprecipitation assay with anti-DC-SIGN antibodies. In addition, we found that anti-DC-SIGN (neutralizing antibodies against DC-SIGN) inhibition abolished the potentiating effects of mtDNA on platelet activation, further confirming the critical role of DC-SIGN in mtDNA-mediated platelet activation. We next studied the role of metformin in mtDNA-mediated platelet activation. We found that platelet activation was dramatically decreased following metformin preincubation. Taken together, our study is the first to reveal that the mtDNA released from activated platelets can act as a cellular damage factor and subsequently induce platelet activation may via a DC-SIGN-dependent pathway. We next considered to confirm and assure mtDNA induces platelet activation through DC-SIGN with gene knockout approaches. Our work presented here is consistent with other recent findings and suggests possible mechanism(s) for metformin’s role in reduced mortality and diabetes-associated thrombotic complications, related to inhibition of platelet activation, prevention of mitochondrial dysfunction, and reduction in the release of free mtDNA.

As of 2014, DM was diagnosed in more than 20 million adults in the United States and likely remained undiagnosed in millions more. Thrombotic cardiovascular events, in which platelets have essential functions, will be a likely cause of death in an estimated 65% of diabetic patients. Although aspirin is most commonly used to prevent and treat heart attacks and strokes in clinical practice, previous work has shown that 10% to 40% of diabetic patients are biochemically resistant to this drug. Due to this important concern, a better understanding of and new therapies to target the underlying mechanism for platelet dysfunction must be found^1^,^2^,^3^,^4^. Our findings suggest that metformin could be a promising lead as a new class of antiplatelet agents that are highly effective at inhibiting platelet activation *in vitro* and *in vivo*.

In conclusion, the research presented here has established a novel therapeutic strategy for platelet abnormalities in DM and a general strategy to prevent cardiovascular complications. Future studies will be required to develop new antiplatelet drugs that are potentially more effective without bleeding risk and as possible treatments for metabolic syndrome. Furthermore, our team will evaluate whether or not metformin also inhibits anionic phospholipid exposure, microvesicle release, and other damage-associated molecular pattern molecules (DAMPs) such as high-mobility group box 1 (HMGB1) in the future. We will use other pharmacological and knockout approaches to confirm and assure mtDNA induces platelet activation through DC-SIGN, and research DC-SIGN downstream signaling events, and consider other receptors that might be implicated in mtDNA dependent platelet activation e.g. TLR9. On the basis of our studies, we now propose that drugs targeting platelet mtDNA may be effective against platelet dysfunction and thrombosis. These mechanistic studies provide new insights into platelet abnormalities to inhibit thrombosis. Beyond the well-recognized pivotal role in thrombosis and hemostasis, platelets also play a critical role in inflammatory and infectious diseases, and increasing evidence indicates that bacterial infection predisposes for atherosclerosis and thrombotic events^26^. Several previous studies provided support that mtDNA is also involved in immune and inflammatory responses and their related diseases^36^,^37^,^38^,^39^. In this study, we reported that mtDNA is released from activated platelets and acts as an ‘agonist’ to induce platelet activation and thrombosis. To our knowledge, this study is the first to report that mtDNA may play a connecting role in thrombosis and inflammation/immunity during bacterial infection and, thus, is an important link to study in elucidating the underlying mechanisms that thrombotic events play in inflammation, infection, cancer, DM, etc.

## Materials and Methods

### Animal models and diets

Male Type 2 diabetic rats (type 2 DM), Sprague-Dawlecy (SD) rats and C57/BL6 mice were obtained from the West China Hospital Experiment Animal Center (Chengdu, China). The rats were maintained in an animal house with a constant temperature of 25 °C and a 12/12-hour of light/dark cycle. Food and water were provided ad libitum. All animals were treated according to the experimental protocols approved by the Bioethics Committee of West China Center of Medical Sciences. Diabetic rats were induced by combination of high-fat diet-fed and low-dose streptozotocin (STZ, 45 mg/kg, ip) injection (Final fasting blood glucose level: Control 4.8 ± 0.8 mmol/L; Diabetic 16.4 ± 2.2 mmol/L)^40^. All animal studies included 9–10 rats or mice per group, aged 9–12 weeks at the time of study. Animals were randomly allocated to control groups, model groups, and treatment groups. Metformin was administrated orally to animals at 200 mg/kg twice daily for seven days. Aspirin was administrated at 15 mg/kg in the same manner. Ferric chloride induced carotid arterial injury and venous thrombosis experiments were conducted according to the previously described methods with a minor modification^25^. Briefly, a piece of filter paper (2 mm × 10 mm) pre-saturated with 3 μL of 1 mM FeCl_3_ solution (arterial) or 10 mm × 10 mm paper pre-saturated with 20 μL of 2 mM FeCl_3_ solution (venous) was placed on the arterial or venous cava. Blood flow was monitored continuously for 90 minutes or until stable occlusion occurred, at which time the experiment was terminated. Stable occlusion was defined as the time at which blood flow remained absent for ≥10 minutes. Finally, weight and length were measured. Inferior vena cava ligation experiments were performed as described under aseptic conditions^41^. Briefly, rats were anesthetized with 5% chloral hydrate (0.7 mL/100 g) and placed in a supine position. A midline laparotomy was performed, and then intestines were exteriorized and sterile saline was applied to prevent drying during the whole procedure. The inferior vena cava (IVC) was ligated by a 7.0 polypropylene suture below the renal veins for obtain complete blood stasis. After surgery, peritoneum and skin were closed and the animal was allowed to recover. After 48 hours, the IVC was harvested and weighed for analysis. Pulmonary thromboembolism was induced by a method as described^42^. The number of dead or paralyzed mice was recorded and the percentage of protection was calculated. The bleeding time was measured by a previously described method^25^,^42^.

### Chronic-type gastric ulcer

Male, C57/BL6 mice (20 ± 3 g) were administrated orally metformin 200 mg/kg twice a day for 60 days. Aspirin was administrated 15 mg/kg in the same way, and mice were sacrificed on the 61^st^ day. The stomach was examined by an observer unaware of the treatments and any macroscopically visible ulcers were measured with calipers. An ulcer area (in mm^2^) was calculated for each stomach. Samples of macroscopically normal and damaged tissue were excised, fixed in neutral buffered formalin, and processed by routine techniques for subsequent histological evaluation. The small intestine was examined for signs of injury^32^.

### Platelet preparation

Washed platelets were prepared by differential centrifugation as described previously^33^,^41^,^43^. Briefly, venous blood samples were collected in citrate by standard venous puncture from rats, and the first 2 mL of blood was discarded to avoid artificial activation. Whole blood was centrifuged (160 g for 10 min) in the presence of PGI_2_ (1 mM) to obtain platelet-rich plasma (PRP). Platelets were subsequently pelleted by centrifugation (1000 g for 5 min). The platelet pellet was then suspended in a washing buffer containing Tyrode’s buffer, 10% of acid-citrate-dextrose solution, 2 mM EDTA and 1 mM PGI_2_ and was centrifuged again. Final samples were resuspended in modified Tyrode buffer. Platelet purity was confirmed by flow cytometric measurement of CD41a expression.

### Platelet adhesion under flow conditions assay

Platelets were incubated at 37 °C for 6 h in the absence or presence of metformin (1 mM), and stained with phalloidin (200 nM) (Sigma-Aldrich) for 30 min at 37 °C as described. A collagen-coated coverslip (Neuvitro) was mounted on a custom-made flow chamber (Chamlide CF; Live Cell Instruments). The fluorescently labeled platelets were then perfused over a matrix of collagen at 150 s^−1^ using a syringe pump (Harvard Apparatus). Non-adherent platelets in the chamber were washed with PBS. Adherent platelets were fixed with cold 4% paraformaldehyde for 15 min and then washed with PBS. The perfusion was live-monitored with a fluorescence microscope (Nikon TE-2000S; Nikon, Melville, NY, USA) equipped with a Nikon DS-2MBWc-U1 CCD camera (Nikon)^34^,^43^,^44^.

### P-selectin and integrin αIIbβ3 assay

After treatment (*in vitro*: metformin: 1 mM, 6 h, 37 °C; aspirin: 0.1 mM, 6 h, 37 °C; mtDNA: 40 ng/μL, 6 h, 37 °C. *In vivo:* metformin: 200 mg/kg twice a day for 7 days; aspirin: 15 mg/kg twice a day for 7 days; mtDNA: 25 μg/kg twice a day for 7 days), platelets were incubated with PE-CD62P (Sigma, USA) or PAC-1-FITC (Sigma, USA) for 30 min in the dark, and reaction was stopped by adding ice-cold PBS. The P-selectin and integrin-αIIbβ3 expression on the platelets was measured at 585 nm (FL2) and 530 nm (FL1) by using flow cytometer (Becton - Dickinson, San Jose, CA, USA). In selected experiments platelets were pre-incubated for 0.5 h at 37 °C with neutralizing antibodies against DC-SIGN (120507; R&D Systems, USA, 25 μg/mL) or DNase I (Genentech, USA, 20 μg/mL)^33^,^45^.

### Lipid peroxidation, ROS, Ca^2+^ and ATP assay

Platelets were incubated with metformin (1 mM) for 6 h at 37 °C. Subsequently, platelets were loaded with CM-H_2_DCFDA (General Oxidative Stress Indicator, Thermo Fisher Scientific, USA) or MitoSOX™ Red Mitochondrial Superoxide Indicator (Thermo Fisher Scientific, USA) at room temperature in the dark and measured by a fluorescence microplate reader (Aynergy Mx, BioTek, USA). Lipid peroxidation of platelets was assayed using a commercial kit (BODIPY^®^ 581/591 C11, Thermo Fisher Scientific, USA). The levels of cytosolic calcium were analyzed by flow cytometer using Fluo-3-AM (Sigma, USA) as a probe, and ATP levels were determined using a commercial kit (Sigma, USA)^15^,^35^,^46^,^47^.

### Complex I assay

Platelets were incubated with metformin (1 mM) for 6 h at 37 °C. Complex I (NADH-ubiquinone 1 reductase) levels were assessed with MitoCheck^®^ Complex I Activity Assay Kit (Item No 700930, Cayman Chemical, USA) (Abcam, USA)^35^,^46^.

### Mitochondrial membrane potential assay

Treated platelets implanted in a 48-well plate were incubated with JC-1 solution (10 μg/mL) at 37 °C for 15 min. Then, the platelets were harvested and resuspended in 0.2 mL of Tyrode’s buffer. The Δψ of labeled platelets was measured by a confocal microscope (Nikon ECLIPSE Ti, Japan)^35^,^46^.

### Mitochondrial routine respiration assay

The effect of metformin on mitochondrial respiration in platelets was assay as described previously^35^. Briefly, after stabilization of routine respiration in MiR05 medium, 1 mM metformin, 12.5 nM rotenone, or either vehicle was added. Oligomycin (1 μg/mL), ATP-synthase inhibitor was added to assess LEAK respiration. The maximal capacity of electron respiratory chain using endogenous substrates (ETS) was measured by application of FCCP. Respiration was sequentially blocked by rotenone (the complex I inhibitor, 2 μM), antimycin (the complex III inhibitor, 1 μg/mL) and sodium azide (the complex IV inhibitor, 10 mM) to assess residual oxygen consumption.

### Transmission electronic microscopy imaging of platelets and mitochondria assay

Washed platelets were prepared treated with and without metformin (1 mM, 6 h) and activated or not by ADP, then were fixed with 2.5% glutaraldehyde for at least 24 h and processed for standard dehydration. Briefly, samples were first washed (3 × 10 min) with Tyrode’s buffer then fixed with osmium tetroxide (1% in sodium cacodylate buffer) for 90 min. Samples were washed again (3 × 10 min) with Tyrode’s buffer and subsequently processed for alcohol dehydration steps (50, 70, 95 and 100% EtOH, 10 min each steps). Samples were then dipped in 100% EtOH for 40 and 10 min respectively, and air-dried overnight. Samples were then coated with palladium and observed with a JEOL 6360LV transmission electron microscope (Tokyo, Japan)^18^,^27^,^48^.

### *In vitro* blood clot dissolution assay

Dissolution of blood clot assay *in vitro* was measured as described with slight modification. In brief, venous blood of rats was gathered into a dry Petri dish and clotted at room temperature. The blood clot was cut into 100 ± 20 mg weight slices after 24 h. Subsequently, these clot slices were put into a 12-well plate (one slice per well) and incubated with or without metformin (metformin: 1 mM, 6 h, 37 °C). The dissolution rate of blood clot was calculated and expressed as: Dissolution rate (%) = [(W1–W2)/W1]*100; where W1 represents clot weigh, and W2 indicates the residual clot weight^49^.

### Hematological parameters

The whole blood was collected by cardiac puncture and treated by different anticoagulants: 3.8% trisodium citrate (9:1, v/v), 10% EDTA (9:1, v/v), and heparin. Platelet-rich plasma (PRP) was obtained by centrifugation at 100 g for 10 min. Platelet-poor plasma (PPP) was prepared by centrifugation at 1000 g for 10 min. Thrombelastogram was measured by thrombelastography (Haemoscope, USA). Blood coagulation factor was measured by a kaolin-activated one-stage partial thromboplastin time method using mice Factor plasma (George King Biomedical). An automated hematology analyzer was used to evaluate the viscosity of those samples. Platelet counts (PLT), mean platelet volume (MPV), immature platelet fraction (IPF), plateletcrit (PCT) and platelet distribution width (PDW) of blood samples were measured by automatic blood cell analyzer (SYSMEX SF-3000, Japan). The platelet aggregation assay was performed at 37 °C using a Lumi-Aggregometer (Chrono-log). Aggregation was initiated by the addition of ADP solution (final concentration, 20 μM), thrombin (0.1 U/mL) and arachidonic acid (AA: 900 μM) *in vivo* (pre-treated with 400 mg/kg/d metformin, 50 μg/kg/d mtDNA or control buffer for 7 d) and *in vitro* (pre-incubated with 1 mM metformin, 40 ng/μL or control buffer for 6 h at 37 °C), and recorded for 5 min. In selected experiments platelets were pre-incubated for 0.5 h at 37 °C with neutralizing antibodies against DC-SIGN (120507; R&D Systems, USA, 25 μg/mL)^41^,^33^,^49^,^50^,^51^.

### mtDNA preparation

The mtDNA was isolated from platelets using a commercial kit (Mitochondria DNA Isolation Kit, Biovision), using western blotting confirm the absence of non-mitochondrial proteins to demonstrate the purity of the mtDNA preparations, and using consistent approaches in this study. Quantification of mtDNA was determined by qPCR assay using a PRISM 7300 sequence detection system (Applied Biosystems)^18^.

### mtDNA quantification *in vitro*

Platelets were isolated from the blood of rats and purity was confirmed by flow cytometric measurement of CD41a expression. Then platelets treated with 1 mM metformin for 6 h at 37 °C. After centrifugation at 700 g for 10 min to remove the pellet (platelet), extracellular mtDNA was isolated from the supernatant using a Mitochondrial DNA Isolation Kit (Catalog K280-50, Biovision, USA), using western blotting confirm the absence of non-mitochondrial proteins to demonstrate the purity of the mtDNA. The concentration of mtDNA was determined by a spectrophotometer (Nanodrop2000, Thermo) or qPCR assay using a PRISM 7300 sequence detection system (Applied Biosystems)^18^,^36^,^50^,^52^.

### mtDNA quantification *in vivo*

Seven days after treatment with 400 mg/kg/d metformin, rats were sacrificed and blood was collected, and mtDNA was extracted with Mitochondrial DNA Isolation Kit (Catalog K280-50, Biovision, USA) for analysis, and confirm the purity of the mtDNA. mtDNA level was measured by qPCR assay using a PRISM 7300 sequence detection system (Applied Biosystems) as previously described^18^,^36^,^50^,^52^. The primer sequences were rat NADH dehydrogenase 1 gene (mtDNA): forward CGCCTGACCAATAGCCATAA, reverse ATTCGACGTTAAAGCCTGAGA. All samples were measured with standards at the same time.

### DC-SIGN expression assay

Platelets were resuspended in Tyrode’s buffer, then treatment with or without mtDNA (mtDNA: 40 ng/μL, 6 h). Immunoprecipitation of protein assemblies was performed by incubation of these pools with purified and identified monoclonal antibodies against DC-SIGN (MR-1, Santa Cruz Biotech, USA), and then capture of the immune complexes on Protein G superparamagnetic Microbeads (Miltenyi Biotec, Germany) as described previously^22^. Immunoprecipitates were subjected to Western blot using polyclonal antibodies against DC-SIGN. Immunoprecipitated with Streptavidin-agarose (Sigma Aldrich, USA), and then immunoprecipitated material was analyzed by SDS-PAGE, Western blot with antibodies specific for DC-SIGN.

### mRNA Levels of *Nox2, Gpx1, Sod1* assay

Levels of mRNA for *Nox2*, *Gpx1*, *Sod1*, and *18S* were measured by quantitative real-time PCR as described previously^33^. First, isolating total RNA from washed platelets with Trizol reagent (Invitrogen, Carlsbad, CA). Reverse-transcribed cDNA was incubated with PCR primers, 6-carboxy fluorescein-labeled probes (Applied Biosystems) and TaqMan Universal PCR mix at 50 °C for 2 minutes and then at 95 °C for 10 minutes. After that, 40 cycles of 95 °C for 15 seconds and 60 °C for 1 minute. Using the comparative threshold cycle (ΔΔCT) method to quantification with values normalized of 18S and expressed relative to levels in platelet without metformin preincubation. Platelet purity was confirmed by flow cytometric measurement of CD41a expression^33^.

### Western blot analysis

Washed platelets were incubated with metformin for 6 h. The platelet were harvested and lysed using lysis buffer, after that the solution was centrifuged. After protein concentrations were determined, individual platelets lysates were separated with sodium dodecyl sulfate polyacrylamide gel electrophoresis (10% gel, SDS–PAGE), subsequently, platelets were transferred onto nitrocellulose membranes. The target proteins in the membranes was blocked with 5% nonfat milk, and probed with anti-caspase 3 (Cell Signaling Technology, Danvers, USA), overnight. The membranes were incubated with peroxidase-conjugated secondary antibody for 2 h. and then visualized with ECL Western Blotting Detection Reagents (Amersham Pharmacia Biotech)^53^.

### Statistical analysis

The data are expressed as mean ± SD. Multiple comparisons were made by appropriate analysis of variance, whereas individual group samples were compared by Student’s *t-test*. In all cases *P* values were two tailed and *P* values < 0.05 were considered to indicate significance.

## Additional Information

**How to cite this article**: Xin, G. *et al*. Metformin Uniquely Prevents Thrombosis by Inhibiting Platelet Activation and mtDNA Release. *Sci. Rep*. **6**, 36222; doi: 10.1038/srep36222 (2016).

**Publisher’s note:** Springer Nature remains neutral with regard to jurisdictional claims in published maps and institutional affiliations.

## Supplementary Material

Supplementary Information
