# Supplementary material for: Metformin Uniquely Prevents Thrombosis by Inhibiting Platelet Activation and mtDNA Release
Source: Sci Rep. 2016 Nov 2;6:36222. doi: 10.1038/srep36222 (PMC5090250; doi:10.1038/srep36222)
Supplement: Supplementary Information [file srep36222-s1.doc]

**Metformin Uniquely Prevents Thrombosis without Increased Bleeding Risk by Inhibiting Platelet Activation and mtDNA Release**

Guang Xin^1,2^, Zeliang Wei^1^, Chengjie Ji^3^, Huajie Zheng^1,4^, Jun Gu^5^, Limei Ma^1^, Wenfang Huang^3^, Susan L. Morris-Natschke^2^, Jwu-Lai Yeh^6^, Rui Zhang^1^, Chaoyi Qin^5^, Li Wen^1,4^, Zhihua Xing^1^, Yu Cao^7^, Qing Xia^4^, Yanrong Lu^8^, Ke Li^1^, Hai Niu^1,9*^, Kuo-Hsiung Lee^2,10*^ & Wen Huang^1*^

**Supplementary Information**


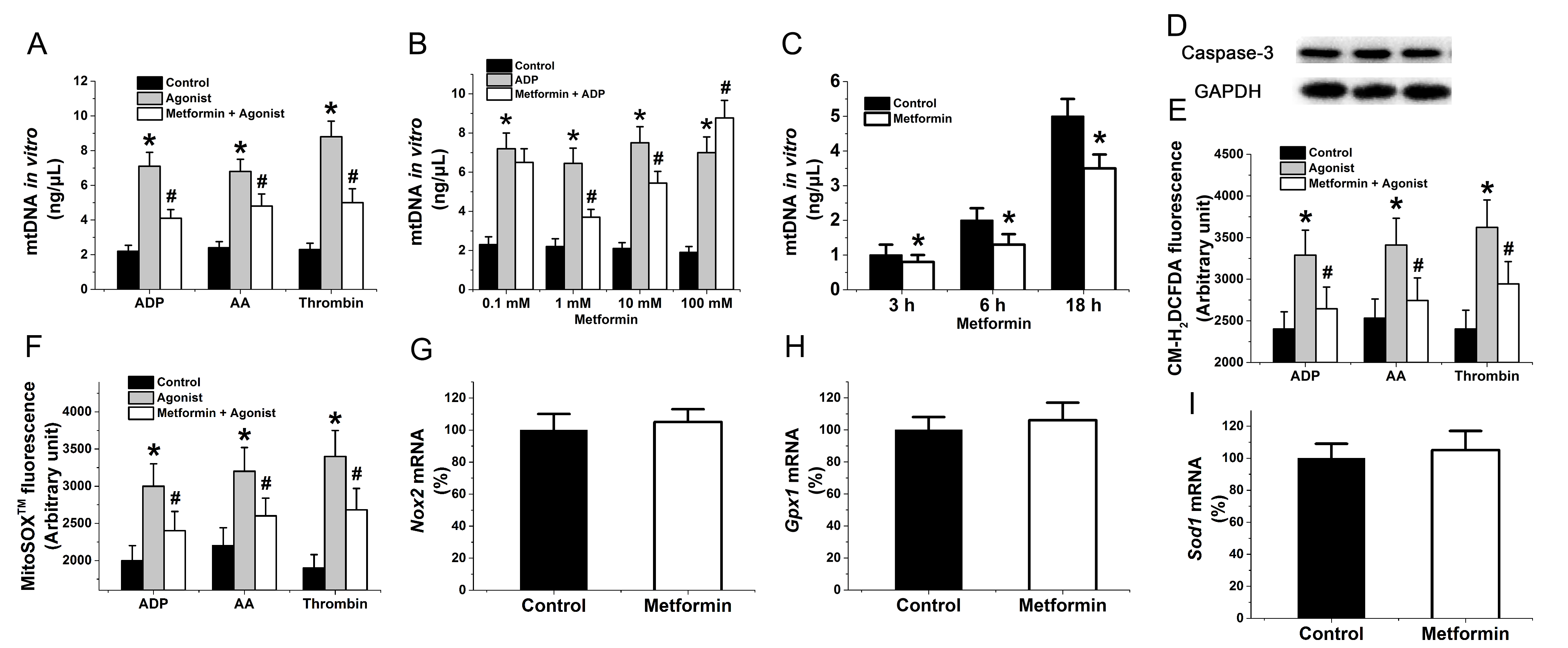


**Supplementary figure 1.** (**A**) Metformin inhibited mtDNA release from ADP, AA, thrombin-activated platelets (metformin: 1 mM, 6 h). (**B**) The dose effects of metformin on mtDNA release from ADP-activated activated platelets (metformin: 6 h). (**C**) The effects of metformin on mtDNA release from resting platelet (without agonist activation) (metformin: 1 mM). (**D**) The expression of Caspase-3 in platelets was examined with or without metformin treatment (metformin: 1 mM, 6 h). (**E**, **F**) Metformin inhibits the production of general ROS (**E**) and mitochondrial ROS (**F**) from ADP, AA, thrombin-activated platelets (metformin: 1 mM, 6 h). (**G**-**I**) Metformin has insignificant influences on NADPH oxidase, glutathione peroxidase-1, and superoxide dismutase in platelets. Platelet mRNA levels of *Nox2* (**G**), *Gpx1* (**H**), and *Sod1* (**I**) with metformin preincubation (metformin: 1 mM, 6 h) were measured by real-time polymerase chain reaction. Values were normalized to *18S* mRNA and are expressed as percent of the control values observed without metformin preincubation. Values are mean ± SD. *n* = 9-10. **P* < 0.05 vs control, *^#^P* < 0.05 vs Agonist.


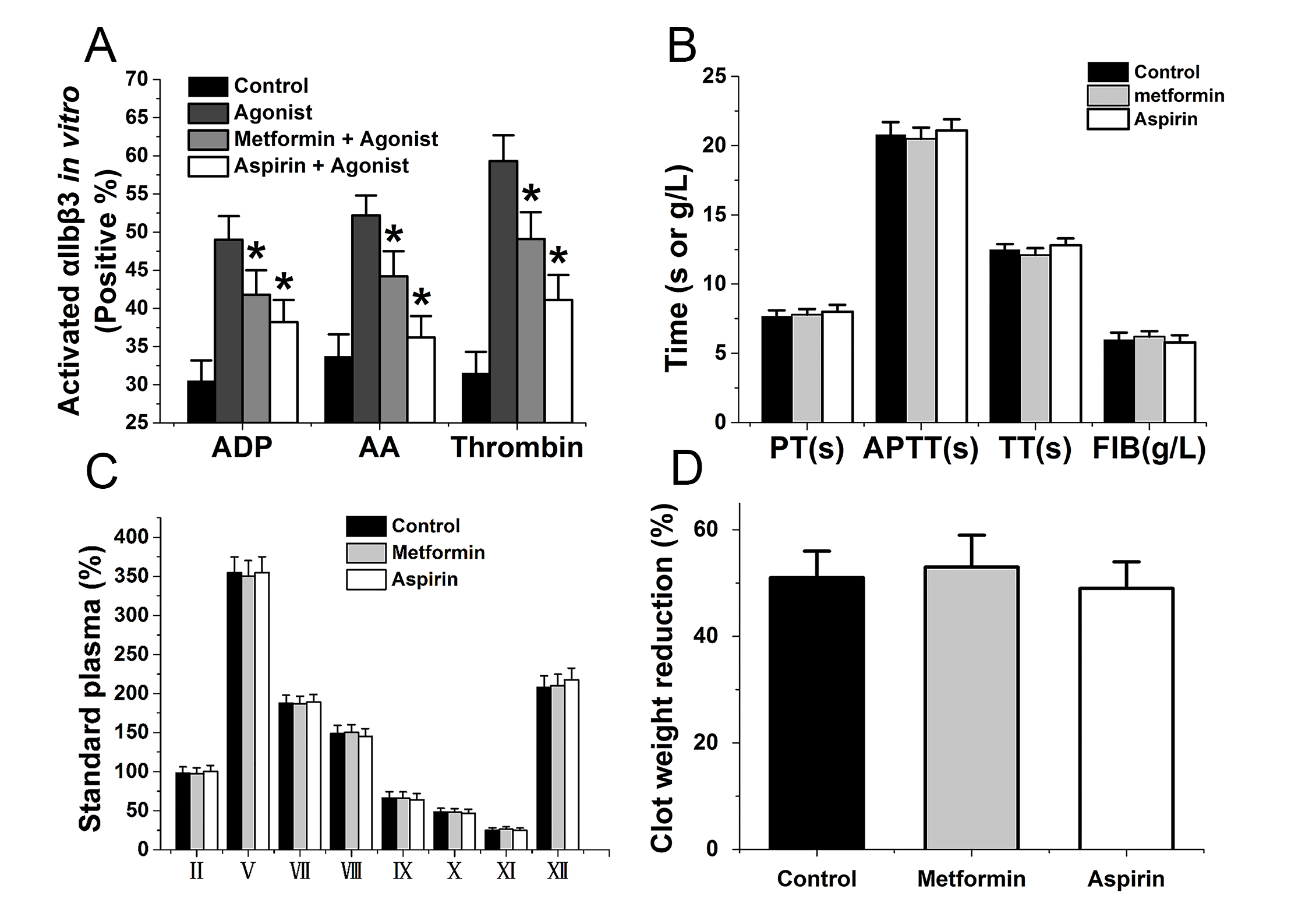


**Supplementary figure 2.** (**A**) Metformin significantly suppressed αIIbβ3 expression of platelets activation induced by ADP, AA and thrombin (metformin: 1 mM, 6 h). (**B**) Metformin has insignificant influences on coagulation factor and thrombolysis system. (**C**, **D**) Effects of metformin treatment in rats (metformin: 400 mg/kg/d, 7 d) on PT, APTT, TT, FIB, coagulation factors (**C**), and clot weight reduction (**D**). Data are expressed as mean ± SD. *n* = 9-10. **P* < 0.05 vs control.


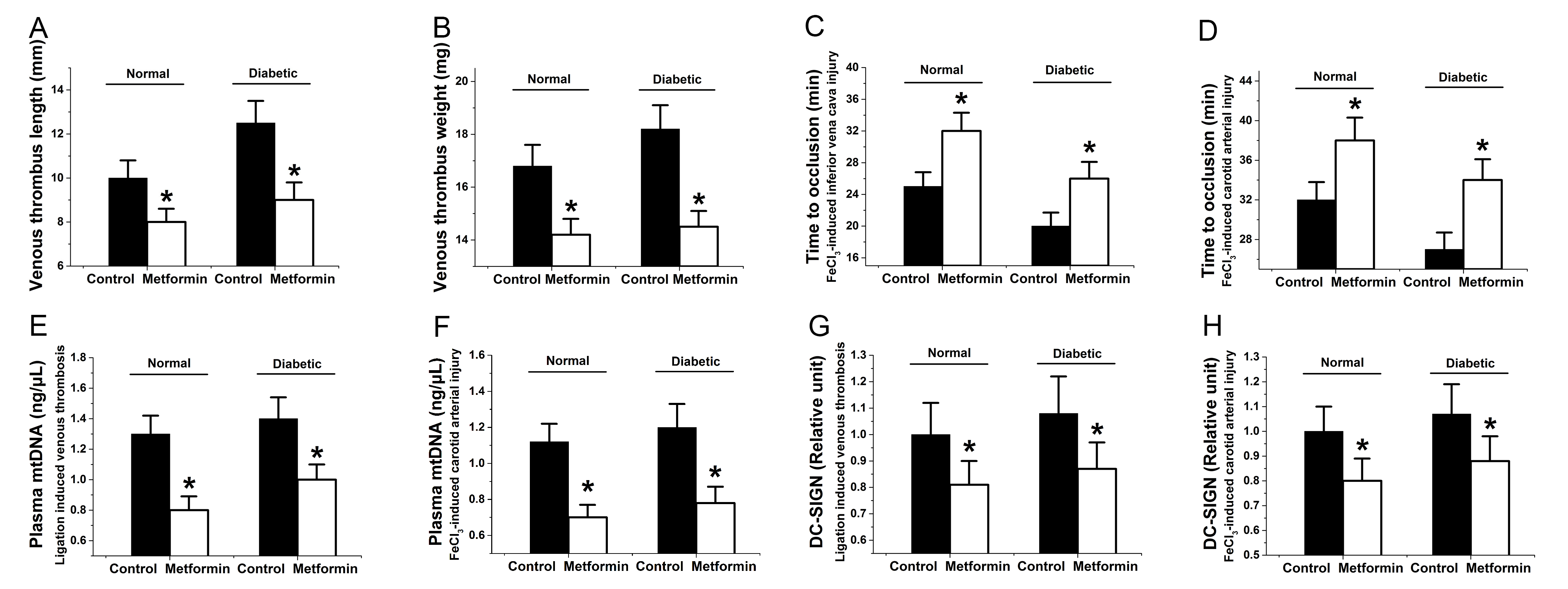


**Supplementary figure 3.** Metformin inhibits formation of FeCl_3_-induced inferior vena cava thrombosis in animals. (**A**, **B**) decreases length and weight of FeCl_3_-induced inferior vena cava thrombus in diabetic and normal rats (metformin: 400 mg/kg/d, 7 d). (**C**, **D**) The average time to occlusive thrombosis in FeCl_3_-induced inferior vena cava injury and carotid arteries injury was longer in metformin treatment (metformin: 400 mg/kg/d, 7 d) diabetic and normal rats compared with no treatment. (**E**, **F**) Metformin decrease the amounts of plasma mtDNA in partial inferior vena cava ligation induced venous thrombosis and FeCl_3_-induced carotid arterial thrombosis. (**G**, **H**) Metformin reduces platelet DC-SIGN expression in in partial inferior vena cava ligation induced venous thrombosis and FeCl_3_-induced carotid arterial thrombosis. Data are expressed as mean ± SD. *n* = 9-10. **P* < 0.05 vs control.

**Supplementary Table 1.** Metformin improves blood viscosity in both normal and diabetic rats (metformin: 400 mg/kg/d, 7 d). Data are expressed as mean ± SD. *n =* 9-10. **P* < 0.05 vs control.

|  | Control- normal-type | Metformin- normal-type | Control- diabetic-type | Metformin- diabetic-type |
| --- | --- | --- | --- | --- |
| Blood [shear rate](http://dict.cnki.net/dict_result.aspx?searchword=切变率&tjType=sentence&style=&t=shear+rate) 1 | 43.22 ± 3.02 | 34.04 ± 2.98^*^ | 46.43 ± 3.10 | 37.76 ± 3.15^*^ |
| Blood [shear rate](http://dict.cnki.net/dict_result.aspx?searchword=切变率&tjType=sentence&style=&t=shear+rate) 5 | 16.88 ± 0.98 | 13.94 ± 1.09^*^ | 20.54 ± 1.21 | 14.68 ± 0.87^*^ |
| Blood [shear rate](http://dict.cnki.net/dict_result.aspx?searchword=切变率&tjType=sentence&style=&t=shear+rate) 50 | 7.32 ± 0.40 | 6.61 ± 0.49^*^ | 9.45 ± 0.56 | 6.81 ± 0.31^*^ |
| Blood [shear rate](http://dict.cnki.net/dict_result.aspx?searchword=切变率&tjType=sentence&style=&t=shear+rate) 100 | 6.35 ± 0.23 | 5.83 ± 0.31^*^ | 8.45 ± 0.19 | 5.65 ± 0.20^*^ |
| Blood [shear rate](http://dict.cnki.net/dict_result.aspx?searchword=切变率&tjType=sentence&style=&t=shear+rate) 200 | 6.40 ± 0.22 | 5.30 ± 0.51^*^ | 6.70 ± 0.10 | 5.08 ± 0.32^*^ |
| [Plasma viscosity](http://dict.cnki.net/dict_result.aspx?searchword=血浆粘度&tjType=sentence&style=&t=plasma+viscosity) | 1.30 ± 0.22 | 1.22 ± 0.26^*^ | 1.45 ± 0.18 | 1.31 ± 0.20^*^ |
| Compression volume | 0.45 ± 0.10 | 0.46 ± 0.09 | 0.53 ± 0.15 | 0.48 ± 0.14 |
| [Erythrocyte sedimentation rate](http://dict.cnki.net/dict_result.aspx?searchword=血沉&tjType=sentence&style=&t=erythrocyte+sedimentation+rate) | 2.02 ± 0.21 | 2.00 ± 0.19 | 2.33 ± 0.17 | 2.10 ± 0.25 |
| High shear viscosity | 4.38 ± 0.22 | 4.03 ± 0.25^*^ | 4.58 ± 0.17 | 3.88 ± 0.26^*^ |
| Low shear viscosity | 33.18 ± 1.03 | 27.82 ± 1.20^*^ | 36.18 ± 0.84 | 29.82 ± 1.12^*^ |
| Erythrocyte sedimentation rate-K value | 8.05 ± 0.42 | 8.46 ± 0.57 | 8.60 ± 0.32 | 7.66 ± 0.40^*^ |
| [Erythrocyte aggregation index](http://dict.cnki.net/dict_result.aspx?searchword=红细胞聚集指数&tjType=sentence&style=&t=erythrocyte+aggregation+index) | 7.58 ± 0.22 | 7.42 ± 0.25 | 8.21 ± 0.20 | 7.43 ± 0.28^*^ |
| Blood [low shear reduced viscosity](http://dict.cnki.net/dict_result.aspx?searchword=高切还原粘度&tjType=sentence&style=&t=high+shear+reduced+viscosity) | 93.15 ± 4.03 | 71.34 ± 3.12^*^ | 99.15 ± 4.17 | 82.83 ± 3.24^*^ |
| Blood [high shear reduced viscosity](http://dict.cnki.net/dict_result.aspx?searchword=高切还原粘度&tjType=sentence&style=&t=high+shear+reduced+viscosity) | 9.77 ± 0.52 | 8.86 ± 0.43^*^ | 10.42 ± 0.88 | 8.58 ± 0.42^*^ |
| Erythrocyte deformation index | 0.99 ± 0.11 | 0.98 ± 0.17 | 0.89 ± 0.22 | 0.95 ± 0.20 |
| [Erythrocyte rigidity index](http://dict.cnki.net/dict_result.aspx?searchword=红细胞刚性指数&tjType=sentence&style=&t=erythrocyte+rigidity+index) | 7.50 ± 0.22 | 7.25 ± 0.20 | 8.50 ± 0.28 | 6.55 ± 0.25^*^ |

**Supplementary Table 2.** Metformin has insignificant effect on platelet properties. Platelet count, platelet distribution width, mean platelet volume, thrombocytocrit, platelet ratio, and immature platelet fraction of mice were determined after metformin treatment for 60 days (400 mg/kg/d).

|  | PLT (10^9^/L) | PDW (fL) | MPV (fL) | PCT | P-LCR (%) | IPF (%) |
| --- | --- | --- | --- | --- | --- | --- |
| **Control** | 870 ± 11 | 7.80 ± 0.55 | 7.50 ± 0.41 | 0.36 ± 0.07 | 7.10 ± 0.30 | 0.50 ± 0.06 |
| **Metformin**  400 mg/kg/d | 882 ± 10 | 8.00 ± 0.44 | 7.60 ± 0.58 | 0.38 ± 0.03 | 7.40 ± 0.40 | 0.40 ± 0.14 |
| **Aspirin**  30 mg/kg/d | 850 ± 12 | 7.80 ± 0.40 | 7.40 ± 0.50 | 0.40 ± 0.04 | 7.30 ± 0.35 | 0.50 ± 0.04 |
| PLT: Platelet count, PDW: Platelet distribution width, MPV: Mean platelet volume PLT, PCT: Thrombocytocrit, P-LCR: Platelet ratio, IPF: Immature platelet fraction. | | | | | | |
